# Schools and Diphtheria

**Published:** 1898-12-03

**Authors:** 


### Schools and Diphtheria.

To get the good without the evil, to give the children the best education that the schools can provide, and at the same time to avoid infecting them with diseases which it is to be feared they too often catch from their fellow-pupils, is a subject worthy of the most careful consideration, and one which demands the most loyal co-operation on the part of the educational and sanitary authorities. At the present time, unfortunately, there seems to be a certain antagonism between those who have to do with the mind and those whose duty it is to concern themselves about the body, in regard to the relation between schools and diphtheria. According to a report just issued by the Public Health Committee of the London County Council, Mr. Shirley Murphy, medical officer to the county, had pointed out so long since as 1894, that the relatively increased mortality from diphtheria among children at the school-age period of life raised a serious question whether the aggregation of children in schools, which had continuously increased since the passing of the Education Act in 1870, had contributed in any important degree to the prevalence of the disease. Since the date of that memorandum Mr. Murphy has shown this to be the case, and has demonstrated in his annual reports that when the schools of the London School Board are closed for the summer there is a notable decrease in the prevalence of the disease, that the prevalence increases when the schools reopen at the conclusion of the holiday, and that this decrease and subsequent increase are especially marked among persons of school age. In opposition to this conclusion Dr. W. R. Smith, medical officer to the School Board, has presented a report to that body in which, after carefully discussing the whole question, he comes to the conclusion "that school influence as such plays but an unimportant part in the enormous increase of the disease during recent years in London"; and now there comes another report from Mr. Murphy, in which he states that on examining Dr. Smith's report he is unable to find a single point which militates against the views he had already expressed, or which, in his opinion, justifies the conclusion that the part played by schools in connection with the increase of diphtheria is "unimportant." The Health Committee fully concur in the view expressed by Mr. Murphy, that this is a matter urgently needing the attention of the Local Government Board and the Education Department, and the Council has now resolved to communicate with these Departments and the London School Board on the subject.

So here the matter for the present stands. We cannot but feel that some *modus vivendi* ought to be arrived at between the educational and the sanitary authorities which should allow of the best knowledge and experience which is available being utilised for the protection of the children whom we compel to attend the elementary schools. It can no longer be alleged that the holiday diminution of diphtheria is but slight in proportion to the total number of the cases which occur. Moreover, if we bear in mind that each case may serve as a focus of infection from which other cases may arise, and that such foci are being steadily planted throughout a district all the year through, the fact that the occurrence of the holidays can produce any notable diminution in the number of cases, notwithstanding that all the other foci of infection are presumably in full operation, is sufficient, we think, to show how important must be the influence of school attendance on the spread of the disease, acting as it must do continuously month after month.

When, indeed, we consider that the complete implantation of diphtheria in a district is a slow process, taking sometimes two or three years for its accomplishment, it is a very surprising thing to find that a four weeks' holiday makes such an impress on the prevalence of the disease as it does. And, so far from considering that school influence plays "an unimportant part" in the dissemination of diphtheria, we should not be surprised to find that its part is even more important than Mr. Murphy's figures would suggest to the uninitiated. There seems to us to be not the slightest excuse for shirking the result of this investigation. If diphtheria is the infectious thing it is believed to be, it would be the most astonishing thing in the world if it could be shown that school influence had no effect in aiding and encouraging its dissemination. Not only the facts, but all the probabilities, are in favour of the schools being at the bottom of much of the mischief; and if that is so, it is no use our burying our heads in the sand. What we have to do is not to deny, but to prevent. Better ventilation, smaller classes, better discipline, not only in the schools, but in the playgrounds and lobbies, and, above all things, skilled inspection of the children, might do much to prevent the dissemination of disease.

